# Case of Placental Presentation Succeeded by Acute Puerperal Peritonitis

**Published:** 1816-06

**Authors:** Richard Edgell

**Affiliations:** Surgeon, of Bristol.


### *For the London Medical and Physical Journal.*

*Case of Placental Presentation succeeded by Acute Puerperal Peritonitis;* by RICHARD EDGELL, Surgeon, of Bristol.

I WAS called, in great haste, on the 18th of last April, to M. C., the lady of a clergyman. She was lying on the sofa, very faint with the loss of blood. She had been in an ill state of health for many years,—was in the last month of pregnancy. She refused submitting to an examination at that time, but promised she would acquiesce if the flooding should increase. I directed she should confine herself to her bed-room, strictly observing a recumbent position; and ordered the nurse to keep every thing in readiness for immediate delivery. My patient had frequent but no considerable returns of flooding till the 29th, when I was again called in great haste. I perceived there was now no time to be lost, and, with great difficulty, got her placed on the bed; but, finding that she still had an aversion to have any thing done till the *pains* came on, I stated to the husband the propriety of calling in some one else. Mr. C. immediately procured Mr. Richard Smith, from whom I have received throughout (as it will appear to Dr. Kinglake) this *extraordinary* case every assistance he is so well able to afford. We made an examination, and found what we apprehended, viz. a placental presentation. We determined on immediate delivery, which I effected with great ease in a few minutes, the os uteri being fully dilated. The vagina was filled with coagula. No *pain* whatever, consequently no *contraction*, had taken place previous to the introduction of the hand, which was done slowly and with much caution. She was delivered of a full-sized female infant, which is doing well.

The uterus was thrown into unequal action, and formed slightly what is called the hour-glass contraction, which obliged me again to introduce the hand to complete the delivery. An opiate was given, and every thing went on favourably till the 2d of May, when I was called at half-past eight in the morning. I distinctly heard groans as I entered the house. Mr. C. had left her, at twelve at night, perfectly easy, and full as well as child-bed women usually are two days after delivery. In a short time after her husband had left her, Mrs. C. was attacked with severe rigors, and excruciating pain in the abdomen, which continued without intermission. I was unfortunately not sent for till half-past eight in the morning, which allowed the disease eight hours' progress. The attempt to put my hand under the bed-clothes was attended with so much pain, that I desisted.

From

From a large orifice, about twenty-four ounces of blood were drawn, which was very sizy. This occasioned syncope. The pain was much abated. Bowels freely open, from a purgative given the day before. The pulse, which was 120 before the bleeding, immediately sunk to 98. Fifteen grains of calomel were given. At six in the afternoon, the pulse was again at 120, and, as the pain had returned, it was determined in consultation to have recourse to bleeding. About twenty ounces were taken, which was followed with great relief. Mrs. C. remained for a long time in a state of syncope. At ten the pulse was 120; but the patient was easy, and apparently much better.

On the 3d, at half-past eight in the morning, there was rather an increase of the pain; Mrs. C. had slept but little; the bowels had been freely acted on by the calomel. At twelve, as the pain was still distressing, fourteen leeches were applied, after which the abdomen was tolerably easy, when pressed; but there was a most distressing urging to make water. To relieve this, three table-spoonsful of an eight-ounce mixture, containing three grains of Tart. Ant. and one ʒ of Tinct. Opii, were given every three hours. At two o'clock, the irritation at the neck of the bladder being increased, the catheter was introduced, and about two table-spoonsful of water drawn off, accompanied with much flatus from the bladder. An injection with eighty drops of Tinct. Opii was thrown up the rectum. At six the catheter was again introduced, and some water drawn off, with much flatus. The tenesmus was now very distressing, and an enema with half an ounce of Tinct. Opii was thrown up. At nine the tenesmus was much relieved; the patient had had some sleep, with frequent startings, and easily awoke.

4th.—At six in the morning tolerably easy; had slept moderately; head-ache severe. At nine the pulse was 120; skin moderate; introduced the catheter, and drew off a pint and half of water. At two complained of bearing-down pains. At six the tenesmus very severe; pulse 140; tongue covered with a brown fur, but not dry; skin moderate; no pain on pressing upon the abdomen; no motion; drew off water, and injected three ounces of Tinct. Opii as an enema. At ten the bearing-down pains were greatly relieved; pulse fluctuated from 130 to 140, and was weak; countenance sunk; skin much colder than natural, and moist; spasmodic twitchings of the face and hands; she complained much of her throat and head; strength much sunk, and the voice scarcely audible. It was agreed in consultation, at six in the afternoon, as it would be impossible for my friend Mr. Smith to to see her again that night, that I might make use of any means I might deem proper; and seeing our patient in this deplorable state, I thought I should not be discharging my duty if I did not hazard a trial of the rectified spirit of turpentine. I gave her rather better than a fluid ounce, and staid fifteen minutes with her to observe its effects. Her pulse rose somewhat in strength, but not in frequency; the skin, I thought, was warmer. At the expiration of the fifteen minutes I repeated the dose. Mrs. C. was evidently roused by the exhibition of the turpentine; I therefore left directions to have it given every three hours, provided she should be able to swallow it.

5th.—At half-past eight in the morning Mrs. C. was better: she had slept but little; pulse 120; skin more natural; tongue brown, but not dry; easy in every particular, except when she had occasion to pass a stool; she had made water frequently, and with perfect ease, which she attributed (to use her own words) to the blessed medicine the turpentine. We were obliged to discontinue it internally, on account of the diarrhoea it occasioned; but, as she was better in other respects, it was directed to be rubbed upon the abdomen, and three table-spoonsful of chalk mixture with conf. cardiac and opium to be given occasionally. Warm port-wine negus was also frequently given. Mrs. C. continued to get better throughout the day: at half-past ten at night she was better in every respect; the friction was ordered to be repeated.

6th.—At eight in the morning, the patient was much the same as yesterday: there was much debility, with depression of spirits, and slight sickness; pulse 120, but stronger; she had slept well, and had passed four watery motions; a glass of warm mulled wine was given, and a saline effervescing draught, to counteract the sickness; jelly and arrow-root, with a little wine, the chief diet. In the evening the tenesmus of the bladder had returned; an opiate enema was thrown up. Ten at night, better; she had slept, and was cheerful.

7th.—At eight in the morning, pulse 110, and rather weak: there had been five motions during the night; she had slept but little; diet as yesterday. The turpentine friction being repeated, occasioned great pain, and re-produced the dysenteric symptoms. An enema made with starch, containing five ounces of Tinct. Opii, was thrown up in the evening. The breasts are beginning to be troublesome, and were ordered to be drawn.

8th.—Much better. We were received with a smile. Mrs. C. had slept well; pulse 120 in the morning, 110 in the evening; evening; debility appears to be the only complaint, but she evidently requires much management and good nursing.

9th.—As well as yesterday: pulse 100; tongue cleaner; four loose stools in the night; appetite returning. An opiate draught was ordered in the evening.

10th.—Going on well.

*May* 10, 1816.

