# Errata

**Published:** 1816-07

**Authors:** 


ERRATA.

In the last volume, p. 463, line 8 from the bottom, for three *ounces* read three *drams.* P. 464, line 5 from the top, for fluid *ounce* read fluid *dram;* and, at line 5 from the bottom, for five *ounces* read five *drams.* P. 520, for *alteria* read *altera.*

